# Single breath-hold assessment of cardiac function using an accelerated 3D single breath-hold acquisition technique - comparison of an intravascular and extravascular contrast agent

**DOI:** 10.1186/1532-429X-14-53

**Published:** 2012-07-31

**Authors:** Marcus R Makowski, Andrea J Wiethoff, Christian HP Jansen, Sergio Uribe, Victoria Parish, Andreas Schuster, Rene M Botnar, Aaron Bell, Christoph Kiesewetter, Reza Razavi, Tobias Schaeffter, Gerald F Greil

**Affiliations:** 1 King’s College London BHF Centre, Division of Imaging Science, Biomedical Research Centre of Guy’s and St. Thomas’ NHS Foundation Trust, London, UK; 2 Department of Radiology, Charite, Berlin, Germany; 3 Philips Healthcare, Guildford, UK; 4 Radiology Department and Biomedical Imaging Center, School of Medicine, Pontifica Universidad Catolica de Chile

**Keywords:** Cardiovascular magnetic resonance, Cardiac function, Balanced steady-state-free precession (SSFP), Gadofosveset trisodium, Gadopentetate dimeglumine, 32-channel coil, Sensitivity encoding (SENSE)

## Abstract

**Background:**

Cardiovascular magnetic resonance (CMR) is the current gold standard for the assessment of left ventricular (LV) function. Repeated breath-holds are needed for standard multi-slice 2D cine steady-state free precession sequences (M2D-SSFP). Accelerated single breath-hold techniques suffer from low contrast between blood pool and myocardium. In this study an intravascular contrast agent was prospectively compared to an extravascular contrast agent for the assessment of LV function using a single-breath-hold 3D-whole-heart cine SSFP sequence (3D-SSFP).

**Methods:**

LV function was assessed in fourteen patients on a 1.5 T MR-scanner (Philips Healthcare) using 32-channel coil technology. Patients were investigated twice using a 3D-SSFP sequence (acquisition time 18–25 s) after Gadopentetate dimeglumine (GdD, day 1) and Gadofosveset trisodium (GdT, day 2) administration. Image acquisition was accelerated using sensitivity encoding in both phase encoding directions (4xSENSE). CNR and BMC were both measured between blood and myocardium. The CNR incorporated noise measurements, while the BMC represented the coeffiancy between the signal from blood and myocardium [1]. Contrast to noise ratio (CNR), blood to myocardium contrast (BMC), image quality, LV functional parameters and intra-/interobserver variability were compared. A M2D-SSFP sequence was used as a reference standard on both days.

**Results:**

All 3D-SSFP sequences were successfully acquired within one breath-hold after GdD and GdT administration. CNR and BMC were significantly (p < 0.05) higher using GdT compared to GdD, resulting in an improved endocardial definition. Using 3D-SSFP with GdT, Bland–Altman plots showed a smaller bias (95% confidence interval LVEF: 9.0 *vs.* 23.7) and regression analysis showed a stronger correlation to the reference standard (R^2^ = 0.92 *vs.* R^2^ = 0.71), compared to 3D-SSFP with GdD.

**Conclusions:**

A single-breath-hold 3D-whole-heart cine SSFP sequence in combination with 32-channel technology and an intravascular contrast agent allows for the accurate and fast assessment of LV function.

**Trial registration:**

The study was approved by the local research ethics committee (Study No. 07/Q0704/2) and was registered with the Medicines and Healthcare Products Regulatory Agency (MHRA Study No. 28482/0002/001–0001, EudraCTnumber 2006–007042).

## Background

The assessment of left ventricular (LV) volumes and function is of high importance in patients with cardiovascular disease as it has therapeutic and prognostic consequences [1]. Cardiovascular magnetic resonance (CMR) is the gold standard method for the assessment of cardiac function and regional wall motion [1-3]. Currently used CMR techniques require the acquisition of multiple short-axis slices during repeated breath-holds to cover the ventricles from base to apex. For severely ill or non-compliant patients, performing multiple breath-holds can be challenging. Additionally, spatial misalignment of slices can occur if patients do not fully comply with breathing commands. A single breath-hold technique allowing the accurate assessment of cardiac volumes and function would significantly reduce scan time and increase patient comfort.

Recently, 3D time resolved cine steady-state-free precession (SSFP) sequences using sensitivity encoding (SENSE) undersampling were introduced for ventricular functional assessment within a single breath-hold [4]. Other available approaches to accelerate image acquisition include parallel imaging [5] and undersampled reconstruction kt-techniques [6,7]. All these time-accelerated acquisition techniques suffer from a reduction in blood to myocardium contrast, compared to standard multi-slice (M2D) sequences with repeated breath-holds. The reduced blood to myocardium contrast results from limited inflow of blood into the acquisition volume, which covers the complete ventricle [1]. This reduced contrast can hamper a clear delineation of endocardial borders, which is necessary for the precise assessment of left ventricular volumes and function.

A recent study indicated, that the administration of an extravascular contrast agent immediately prior to image acquisition results in an increase in blood to myocardium contrast using a 3D-SSFP sequence [8]. Extravascular contrast agents rapidly redistribute into extravascular tissues, thereby limiting the contrast between myocardium and blood. Additionally, this type of contrast agent has a 5- to 6-fold lower R1 relaxivity at 1.5 T compared to novel intravascular contrast agents [9].

In this study, the following hypothesis was tested: The administration of an intravascular contrast agent in combination with a single-breath-hold 3D-whole-heart cine SSFP sequence using a 32-channel cardiac coil and SENSE undersampling allows a more accurate assessment of the left ventricular function compared to an extravascular contrast agent.

## Methods

### Study population and design

Fourteen patients with congenital heart disease (age: 23 to 43 years; median: 36 years) were prospectively enrolled into this study. All patients were in sinus rhythm. Written informed consent was obtained from all patients. The study was approved by the institutional review board (IRB No. 07/Q0704/2) and was registered with the Medicines and Healthcare Products Regulatory Agency (MHRA Study No. 28482/0002/001-0001, EudraCT number 2006–007042). Only individuals without contraindications to CMR and MR contrast agents were included in this study. Any suspicion of kidney impairment resulted in exclusion from the study. Patients were given a take-home informed consent regarding MR contrast media.

**Figure 1:**
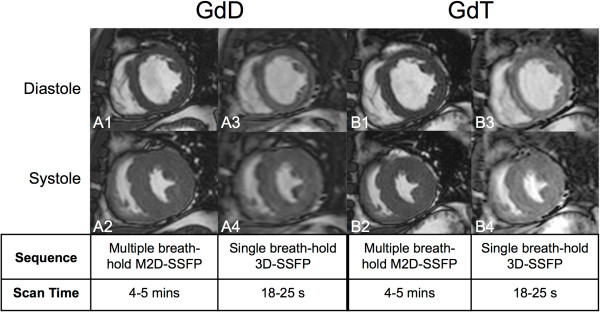
**Representative examples of the left-ventricular mid-portion in end-diastolic (A1, A3, B1, B3) and end-systolic phase (A2, A4, B2, B4) for standard multi-slice 2D cine SSFP sequences (M2D-SSFP).** Examples of single-breath-hold 3D-whole-heart cine SSFP (3D-SSFP) CMR after administration of GdD (A3, A4) and GdT (B3, B4). GdD, Gadopentetate dimeglumine; GdT, Gadofosveset trisodium.

The imaging protocol of this study was designed to provide an intra-individual evaluation of functional left ventricular parameters. The same patient was investigated twice. On day 1, Gadopentetate dimeglumine (GdD, Magnevist®, Bayer Schering Pharma AG, Berlin, Germany; max. 0.2 mmol/kg; max. volume 40 ml) and on day 2 (> 24 hours but within 6 days after administration of GdD) Gadofosveset trisodium (GdT, Vasovist®, Bayer Schering Pharma AG, 0.03 mmol/kg) was injected. The contrast agents were given as part of a first-pass MR angiogram and a subsequent cardiac evaluation, which was clinically indicated. All examinations were performed on a 1.5 T clinical MR scanner (Achieva, Philips Healthcare, Best, Netherlands) equipped with a 32-element cardiac coil (16 anterior and 16 posterior elements). After localization and coil sensitivity reference scans, an interactive scan was used to determine the geometry of the short axis view. Based on these images, planning of standard multi-slice cine 2D SSFP sequences (M2D-SSFP) and single-breath-hold 3D-whole-heart cine SSFP sequence (3D-SSFP) was performed. In all patients, 3D-SSFP sequences were performed between 3–9 minutes after the administration of the respective contrast agent (GdT or GdD). In a subgroup of patients standard M2D-SSFP, associated with multiple breath-holds, was performed for volumetric and functional ventricular assessment (n = 8). M2D-SSFP scans were performed on both days for each patient prior to the administration of the respective contrast agent. Respective imaging parameters for M2D and 3D sequences: Flip angle (degree) 60, 60; TR/TE 1.6/3.2, 1.8/3.5; Field of view (mm) 340x340, 340x340; Spatial resolution (mm) 1.4x1.4, 1.45x1.45; Slice thickness (mm) 10, 10; Number of slices 10–12, 10–12; Number of breath-holds 5–6, 1; Breath-hold duration (s) 11–18, 18–25; Acceleration factor (SENSE acquisition) 2 (AP), 4 (2 AP x 2 RL); Cardiac Phases 20, 15. The 3D cine b-SSFP acquisition (3D SSFP) was accelerated using a SENSE factor of 2 in both phase-encoding directions (anterior posterior [AP] being the first phase-encoding direction, and feet-head [FH] being the second phase-encoding direction) and partial Fourier reconstruction (factor of 0.62) in the first phase-encoding direction. Furthermore 75% of the cardiac phases were acquired (*i.e.* 15 phases), which were interpolated to 20 phases. This results in an overall acceleration factor of 8.6. Image results were compared quantitatively and qualitatively (Figure 1).

### Image analysis

**Figure 2:**
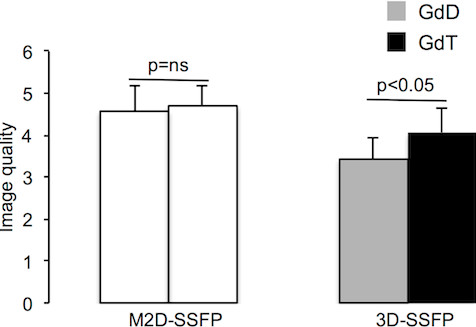
**Image quality for standard multi-slice 2D cine SSFP (M2D-SSFP) and single-breath-hold 3D-whole-heart cine SSFP (3D-SSFP) sequences.** Image quality was significantly (p < 0.05) higher using GdT compared to GdD. GdD, Gadopentetate dimeglumine; GdT, Gadofosveset trisodium.

**Figure 3:**
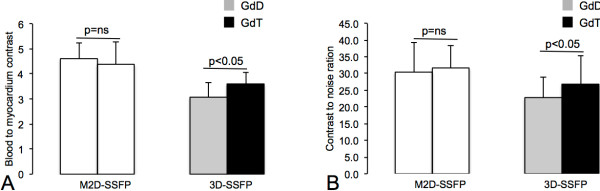
**Blood to myocardium contrast (BMC, A) and contrast to noise ratio (CNR, B) for standard multi-slice 2D cine SSFP (M2D-SSFP) and single-breath-hold 3D-whole-heart cine SSFP (3D-SSFP) sequences.** BMC and CNR were significantly (p < 0.05) higher using GdT compared to GdD.

Image processing was performed using commercially available analysis software (View Forum, Philips Healthcare, Best, The Netherlands). Image data were assessed qualitatively (Figure 2) and quantitatively (Figure 3 and Table 1).

**Table 1:** Left Ventricular Functional Parameters

	**Day 1**		**Day 2**	
	**M2D-SSFP**	**3D-SSFP + GdD**	**M2D-SSFP**	**3D-SSFP + GdT**
ED volume (ml)	147.2±29.9	145.0±39.0	140.2±22.1	143.6±17.6
ES volume (ml)	57.9±19.1	65.6±27.4	59.6±14.4	61.2±11.8
Ejection fraction (%)	60.9±8.6	56.0±11.0	57.7±7.2	57.5±5.8

Left ventricular functional parameters for standard multi-slice 2D cine SSFP (M2D-SSFP) and single-breath-hold 3D-whole-heart cine SSFP (3D-SSFP) sequences. No significant differences (P > 0.05) were found between volume and EF on same day. Values are given as mean ± standard deviation. ED, end diastolic; ES, end systolic; GdD, Gadopentetate dimeglumine; GdT, Gadofosveset trisodium.

### Qualitative image analysis

Consensus reading was performed for image quality scoring by 2 readers (G.F.G, A.B., >10 and >5 years experience in cardiac MR). Prior to the analysis, a trial assessment of 5 separate CMR images of all MR sequences for quality assurance was performed. Subsequently, the two readers analyzed all images independently in a blinded and random order. Disagreements were discussed before a final single grade was given. The image grading system was adopted from McConnell *et al.* (Table 2) [10].

**Table 2:** Image quality scoring system

**Score**	**Description**
1	Poor-image quality: non diagnostic
2	Endocardial border barely or not delineable
3	Endocardial border moderately or partly delineable
4	Endocardial border well delineable
5	Endocardial border excellently delineable

Image quality scoring system adapted from McConnell et al [10].

### Quantitative image analysis

Mean signal intensities (S) of the myocardium and blood pool were measured at end diastole. For assessment of the signal of the myocardium the region of interest (ROI) was placed in the septal myocardium of the mid-ventricle. The signal of blood was defined as the mean signal from a ROI drawn in the center of the left ventricle at enddiastole, excluding the papillary muscles. Noise (N) was determined by the standard deviation in the respective ROIs, as parallel imaging was used (SENSE) [11]. The noise (N) for all measurements was determined by the standard deviation in the respective ROIs, as parallel imaging (SENSE) was used.

The contrast between blood pool and myocardium was calculated as [12]:

(1) $$BMC=\frac{S_{\mathrm{MeanBlood}}}{S_{\mathrm{MeanMyocardium}}}$$

CNR was defined as described in the following equation [13]:

(2) $$CNR=\frac{S_{\mathrm{MeanBlood}}-S_{\mathrm{MeanMyocardium}}}{0.5\cdot \left(N_{\mathrm{MeanBlood}}+N_{\mathrm{MeanMyocardium}}\right)}$$

### Ventricular volume analysis

Volumes derived from single-breath-hold 3D-whole-heart cine SSFP sequences (3D-SSFP) after the administration of GdD and GdT for the evaluation of LV volumes were compared to volumes derived from standard multi-slice 2D cine SSFP sequences (M2D-SSFP, reference standard). In a subgroup of patients standard M2D-SSFP, associated with multiple breath-holds, was performed for volumetric and functional ventricular assessment (n = 8). M2D-SSFP scans were performed on both days in each patient prior to the administration of the respective contrast agent. We included this group, to intra-individually compare M2D-SSFP (as clinical reference standard) to 3D-SSFP scans regarding ventricular function. The four chamber view was used to plan the short-axis stack. The most basal slice was defined when at least 50% of the LV myocardial circumference was visible at end-diastolic and end-systolic short axis slices. The end-diastole and end-systole were defined as the mid-ventricular temporal frame, in which the image showed the largest and smallest LV cavity area. Papillary muscles were excluded from the analysis of the ventricular volume [12]. Measurements were performed by MRM and GFG. Left ventricular volume and ejection fraction were compared between both methods (Table 1).

For interobserver variability two independent observers analyzed 8 of the datasets. For intraobserver variability the same datasets were evaluated twice by the same observer. Datasets were used for the analysis of intraobserver and interobserver variability were randomly chosen for both analysis.

### Statistics

Continuous variables are reported as mean ± standard deviation. Paired and unpaired t-testing was used to compare continuous variables, as appropriate. The agreement between observers regarding image quality grading was assessed by the Wilcoxon signed rank test. A p-value < 0.05 was considered statistically significant. Linear regression analysis was used to assess the volumetric agreement of 3D-SSFP with M2D-SSFP sequences. Bland–Altman analysis was used to assess differences and bias [14].

## Results

Fourteen patients were enrolled into the study protocol. No side effects were correlated to the use of either contrast agent (> 1 year follow-up). There was no significant (p > 0.05) difference in heart rate between the first and second scan session.

### Qualitative image analysis

Image quality of single-breath-hold 3D-whole-heart cine SSFP sequence (3D-SSFP) after the administration of GdD and GdT were compared (Figure 1). GdT improved image quality significantly (p < 0.05) in 3D-SSFP scans compared to GdD (Figure 2).

### Quantitative image analysis

Results from the blood to myocardial contrast analysis (BMC) and contrast to noise ratios (CNR) are shown in Figure 3. After GdT administration, single-breath-hold 3D-whole-heart cine SSFP (3D-SSFP) images demonstrated a significantly (p < 0.05) higher BMC and CNR compared to 3D-SSFP images after GdD administration (Figure 3).

### Left ventricular volume analysis

Left ventricular parameters for all scan groups are shown in Table 1. No significant difference between the M2D-SSFP and respective single-breath-hold 3D-whole-heart cine SSFP sequence (3D-SSFP) scans on the same day were measured for LVEDV, LVESV and LVEF.

**Figure 4:**
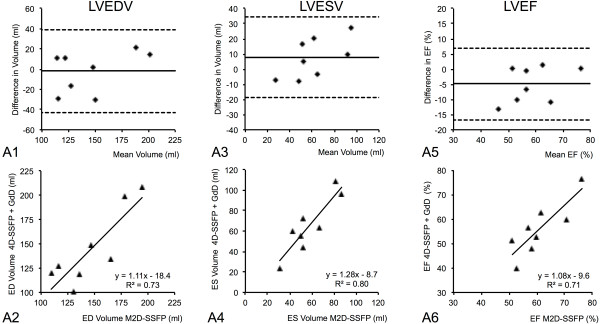
**Bland-Altman plots demonstrating the agreement between standard multi-slice 2D cine SSFP (M2D-SSFP) and single-breath-hold 3D-whole-heart cine SSFP (3D-SSFP) sequences after the administration of Gadopentetate dimeglumine (GdD) with regard to the assessment of LV end-diastolic volume (LVEDV; A1, 2), LV end-systolic volume (LVESV; A3, 4), LV ejection fraction (LVEF; A5, 6).** Bland–Altman plots show a relatively high bias and regression analysis show a moderate correlation using 3D-SSFP with GdD compared to M2D-SSFP as reference standard. Dashed lines equal the 95% limits of agreement. Bias (solid line) equals mean difference between techniques.

**Figure 5:**
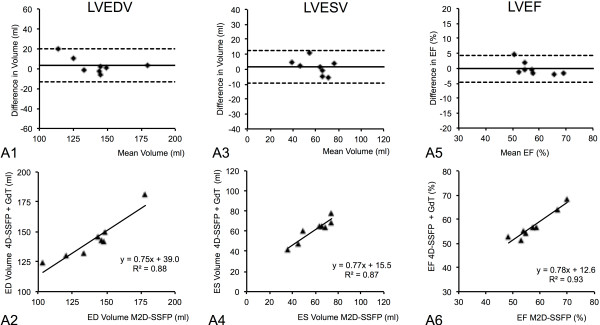
**Bland-Altman plots demonstrating the agreement between standard multi-slice 2D cine SSFP (M2D-SSFP) and single-breath-hold 3D-whole-heart cine SSFP (3D-SSFP) sequences after the administration of Gadofosveset trisodium (GdT) with regard to the assessment of LV end-diastolic volume (LVEDV; A1, 2), LV end-systolic volume (LVESV; A3, 4), LV ejection fraction (LVEF; A5, 6).** Bland–Altman plots show a small bias and regression analysis shows a strong correlation using 3D-SSFP with GdT compared to M2D-SSFP as reference standard. Dashed lines equal the 95% limits of agreement. Bias (solid line) equals mean difference between techniques.

Using 3D-SSFP with GdT, Bland–Altman plots showed a smaller bias (95% confidence intervals LVEF: 9.0 *vs.* 23.7) and a regression analysis showed a stronger correlation with M2D-SSFP as the reference standard (R^2^ = 0.92 *vs.* R^2^ = 0.71), compared to 3D-SSFP with GdD (Figure 4, 5). 95% confidence intervals and means of intra- and interobserver variability are shown in Tables 3 and 4. For intra- and interobserver variability no statistical differences was found comparing the mean functional values (LVEF, LVEDV, LVESV) for 3D-SSFP data with M2D-SSFP reference data, confirming the reproducibility of these techniques.

**Table 3:** Intraobserver Variability

	**95% Confidence interval (ml)**			**Mean difference**		
	**LVEDV (ml)**	**LVESV (ml)**	**LVEF (%)**	**LVEDV**	**LVESV**	**LVEF**
M2D-SSFP	[−7.1; 9.3]	[−9.5; 9.5]	[−6.6; 6.2]	1.1	0.03	−0.2
3D-SSFP + GdD	[−10.2; 8.7]	[−13.8; 11.3]	[−9.2; 9.7]	−0.7	−1.3	0.3
M2D-SSFP	[−7.1; 6.3]	[−6.2; 4.6]	[−5.0; 5.7]	−0.4	−0.8	0.3
3D-SSFP + GdT	[−6.3; 8.4]	[−10.7; 10.3]	[−6.6; 7.7]	1.1	−0.2	0.5

Intraobserver variability for standard multi-slice 2D cine SSFP (M2D-SSFP) and single-breath-hold 3D-whole-heart cine SSFP (3D-SSFP) sequences. 95% Confidence intervals for 3D-SSFP + GdT were narrower compared to 3D-SSFP + GdD. ED, end diastolic; ES, end systolic; GdD, Gadopentetate dimeglumine; GdT, Gadofosveset trisodium.

**Table 4:** Interobserver Variability

	**95% Confidence interval**			**Mean difference**		
	**LVEDV (ml)**	**LVESV (ml)**	**LVEF (%)**	**LVEDV**	**LVESV**	**LVEF**
M2D-SSFP + GdD	[−14.9; 12.4]	[−12.0; 9.0]	[−5.1; 5.9]	−1.3	−1.5	0.4
3D-SSFP + GdD	[−19.4; 21.7]	[−14.2; 12.1]	[−9.2; 12.5]	1.2	−1.1	1.6
M2D-SSFP + GdT	[−9.3; 10.8]	[−9.2; 8.3]	[−6.7; 7.9]	0.7	−0.4	0.6
3D-SSFP + GdT	[−7.9; 12.5]	[−13.2; 10.5]	[−8.8; 11.3]	2.3	−1.4	1.2

Interobserver variability for standard multi-slice 2D cine SSFP (M2D-SSFP) and single-breath-hold 3D-whole-heart cine SSFP (3D-SSFP) sequences. 95% Confidence intervals for 3D-SSFP + GdT were narrower compared to 3D-SSFP + GdD. ED, end diastolic; ES, end systolic; GdD, Gadopentetate dimeglumine; GdT, Gadofosveset trisodium.

### Imaging time

Scan time for single-breath-hold 3D-whole-heart cine SSFP sequences (3D-SSFP) was significantly shorter compared to M2D-SSFP sequences (18-25 s *vs.* 4–5 mins, p < 0.05).

## Discussion

In this prospective study, the administration of an intravascular contrast agent in combination with a single-breath-hold 3D-whole-heart cine SSFP sequence (3D-SSFP) improved image quality significantly. This resulted in a more accurate assessment of LV function compared to the use of an extravascular contrast agent. The use of 32-channel-coil technology and a SENSE factor of 4, enabled imaging with a temporal and spatial resolution comparable to standard multi-slice 2D cine SSFP sequences (M2D-SSFP) [11]. The use of GdT improved the delineation of left ventricular endocardial borders and enabled the accurate quantification of LV volumes and function within one breath-hold. The higher CNR and BMC measured after the administration of GdT, resulted from the higher relaxivity of the contrast agent and its prolonged intravascular half life, compared to the extravascular contrast agent used [15-23]. This study demonstrated that 3D assessment of ventricular function with high accuracy is feasible in a significantly shortened imaging time. Severely ill patients may particularly benefit from shorter scans times, while scanning costs are reduced.

Gadofosveset trisodium reversibly binds to serum albumin with a binding fraction of approximately 85% and therefore predominately remains in the blood pool. High contrast imaging was shown to be feasible up to 60 min after contrast agent administration [15]. The binding to albumin lowers the rate of molecular tumbling of the gadolinium, resulting in a better rotational correlation with water protons. This yields a 5- to 6-fold greater R1 relaxivity at 1.5 T compared with conventional gadolinium contrast agents [9]. With regard to NSF, no cases have been associated with the use of gadofosveset trisodium so far. Due to the greater relaxivity, gadofosveset can be administered at a 3-fold lower dose, while providing greater enhancement from the ventricular blood pool. The usefulness of intravascular contrast agents for the assessment of 3D vascular structures has already been demonstrated [24,25].

A recently published study focused on the direct comparison of non-contrast-enhanced 2D-SSFP sequences *vs.* non-contrast-enhanced 3D acquisitions and gadopentetate dimeglumine enhanced 3D acquisitions [5]. This study demonstrated that the CNR between blood and myocardium in 3D acquisitions increases directly after the administration of an extravascular contrast agent.

The administered contrast agents would also have had an effect on gradient echo (GRE) sequences. GRE profits from shortening the T1-relaxtion time of blood. In particular, GRE is often used to study myocardial function at higher field strength (*e.g.* 3 Tesla). However, a high blood contrast with GRE is only possible through in-flow enhancement in 2D imaging or after administration of a contrast agent. In spoiled GRE the image contrast is proportional to sqrt(TR/2 T1) for the optimal flip-angle and thus profits from shortening T1. However, even for a short T1 relaxation time the overall signal is usually lower than in SSFP imaging, in which case the contrast is proportional to 0.5*sqrt(T2/T1) resulting in a relatively high signal and thus SNR. Although a contrast agent is shortening both the T1 and T2 relaxation time, the T1 effect is usually higher. Therefore, in SSFP a signal increase can also be found after contrast agent administration. The signal enhancement can be calculated for a tissue (*e.g.* blood) for a given contrast agent concentration (assuming the T1 of the blood reduces from 1400 ms to 220 ms). In spoiled GRE the blood signal would increase from 0.035 to 0.09. In SSFP the blood signal would increase from 0.22 to 0.4 (taking also the small T2 shortening into account). Although the relative signal increase is larger in GRE the overall signal amplitude is higher in SSFP imaging resulting in a higher SNR of SSFP in comparison to GRE.

Other potential applications of 3D techniques include pharmacological stress MRI scans of the myocardium. Traditionally, three short axis slices with intra-slice gaps are planned through the LV [26]. Each slice has to be acquired within a separate breath-hold during stress conditions. A 3D approach may offer an attractive alternative, as it is a fast acquisition method, which allows covering both ventricles within one breath-hold with high temporal resolution. The administration of an intravascular contrast agent may be beneficial, as a clearer delineation of the endocardial border is possible, which is especially important for the assessment of regional wall motion. Its use for myocardial perfusion stress testing is currently under investigation.

In this study we could demonstrate, that the single breath-hold assessment of cardiac function using a highly accelerated 3D single breath-hold acquisition technique in combination with a low dose (0.03 mmol/kg) of an intravascular contrast agent allows for the accurate and fast assessment of LV function.

## Conclusion

In conclusion, the use of a 3D-whole-heart cine SSFP sequence in combination with 32-channel technology and an intravascular contrast agent allows for the accurate and fast assessment of LV function within a single breath-hold.

### Study limitations

No conclusion from our data is applicable to patients with arrhythmia. It is also important to note that the volumetric data were acquired at different time points during separate CMR examinations in a resting patient, which may cause slight changes in ventricular volumes. As this technique relies on contrast enhancement after the administration of a contrast agent, it is not applicable in patients with renal impairment. The role of intravascular contrast agents for imaging myocardial delayed enhancement is subject to on-going investigations and has not been addressed in the current study. In case information on myocardial scar imaging is relevant, gadopentetate dimeglumine may be the preferred contrast agent at the moment. However, improved cardiovascular imaging can be provided additional to faster functional assessment of the LV using intravascular contrast agents, which may be very useful in patients with congenital heart disease. Significant flow artifacts in the ventricles were not observed in our patient population during the cardiac cycle as none of our patients had abnormal inflow or outflow of the cardiac chambers.

## Competing interests

The magnetic resonance imaging scanner is partly supported by Philips Healthcare. Andrea Wiethoff is employed by Philips Healthcare. The study was partly supported by Bayer Schering Pharma GmbH. Otherwise there are no financial or other relations that could lead to a conflict of interest.

## Authors’ contributions

Study concepts/study design or data acquisition or data analysis/interpretation, GFG, MRM, AJW, RMB, RR, TS; manuscript drafting or manuscript revision for important intellectual content, all authors; manuscript final version approval, all authors.
